# Aging Triggers an Intestinal Energy Crisis and HDL3 Deficiency Disrupting Gut–Liver Axis Homeostasis

**DOI:** 10.1111/acel.70445

**Published:** 2026-03-18

**Authors:** Yumeng Li, Tongtong Bao, Lumin Gao, Xutong Tian, Junyu Xue, Caike Jin, Shujin Wang, Xin Wu

**Affiliations:** ^1^ State Key Laboratory of Engineering Biology for Low‐Carbon Manufacturing, Tianjin Institute of Industrial Biotechnology Chinese Academy of Sciences Tianjin China; ^2^ KY‐Rubyberries (Fangchenggang) Biotechnoloies Limited Fangchenggang China; ^3^ Center for Obesity and Metabolic Diseases Research, School of Basic Medicine Chongqing Medical University Chongqing China

**Keywords:** aging, gut‐derived HDL3, gut–liver axis, mitochondrial function, NAD^+^, NMN

## Abstract

During aging, decreased intestinal barrier function and its ability to synthesize metabolites are closely associated with various age‐related diseases. However, the mechanism by which impaired intestinal synthesis contributes to gut–liver axis aging remains unclear. This study reveals that aging induces a mitochondrial energy crisis and defective membrane localization of ABCA1, significantly inhibiting the biosynthesis of high‐density lipoprotein 3 (HDL3) in the intestine. Exogenous supplementation with β‐nicotinamide mononucleotide (NMN) restores intestinal NAD^+^ homeostasis, enhances oxidative phosphorylation efficiency, and promotes ATP‐dependent lipid transport, thereby rejuvenating the production of gut‐derived HDL3. Further investigations demonstrate that gut‐originated HDL3 neutralizes lipopolysaccharide (LPS) in the liver and attenuates TLR4‐mediated inflammatory cascades, ultimately ameliorating age‐related liver injury. These findings elucidate a novel mechanism whereby NMN modulates the NAD^+^–mitochondria–ABCA1–HDL3 axis to preserve gut–liver axis function, offering a promising therapeutic strategy for mitigating aging‐related pathologies in this metabolic cross‐talk.

## Graphical abstract

The age‐related mitochondrial energy crisis and the consequent disruption of intestinal HDL3 biosynthesis converge to disrupt the gut–liver HDL3‐LPS/TLR4 axis, compromising hepatic anti‐inflammatory function and driving liver injury, an effect reversible upon NMN administration.

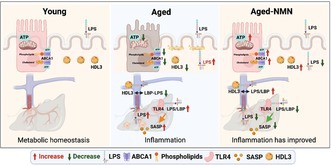

## 1 Introduction

As a complex biological process characterized by the progressive decline of tissue and organ function, aging accompanied by increased susceptibility to age‐related diseases and shortened healthspan (Collado and Serrano 2010; De Miguel et al. 2021; Yu et al. 2021; Babu and Mohanty 2023). A central driver of this metabolic dysregulation is the marked decline in intracellular nicotinamide adenine dinucleotide (NAD^+^) levels (Belenky et al. 2007; Bertoldo et al. 2020; Stromland et al. 2021; Ru et al. 2022). As an essential cofactor in redox reactions, NAD^+^ plays a critical role in cellular energy metabolism, and its depletion directly impairs mitochondrial function, reduces ATP production efficiency, and compromises various energy‐dependent biosynthetic pathways (Bertoldo et al. 2020; Nakagawa and Guarente 2014; Okabe et al. 2020; Rajman et al. 2018; Yoshino et al. 2018).

The gut–liver axis is crucial in maintaining systemic homeostasis, encompassing nutrient absorption, metabolic regulation, and immune defense (Gerner et al. 2018; Schnabl and Brenner 2014; Tripathi et al. 2018). Aging is associated with compromised intestinal barrier integrity, facilitating the translocation of gut‐derived lipopolysaccharide (LPS) and other microbial metabolites via the portal vein to the liver, thereby promoting local and systemic inflammation (Han, Onufer, et al. 2021; Schwabe et al. 2006; Todoric et al. 2020). Recent evidence indicates that gut‐derived high‐density lipoprotein 3 (HDL3) can effectively neutralize LPS and attenuate hepatic inflammation and fibrosis (Tani et al. 2020; Han, Li, et al. 2021; Zheng et al. 2022; Zhao and Gregersen 2015). The synthesis and secretion of HDL3 in enterocytes are known to depend on ATP‐binding cassette transporter A1 (ABCA1)‐mediated lipid efflux, a process highly sensitive to cellular energy status and NAD^+^‐dependent sirtuin signaling pathways (Echesabal‐Chen et al. 2025). However, it remains unclear whether aging impairs HDL3 synthesis and secretion via mitochondrial dysfunction, reduced ATP availability, and diminished ABCA1‐mediated lipid efflux.

β‐nicotinamide mononucleotide (NMN), a direct precursor of NAD^+^, offers a promising strategy for restoring intestinal metabolic function (Irie et al. 2020). After intestinal absorption via the specific transporter SLC12A8 (Grozio et al. 2019; Zheng et al. 2024), NMN significantly elevates intracellular NAD^+^ levels (Das et al. 2018; Irie et al. 2020; Tarantini et al. 2019; Yamamoto et al. 2014; Yoshino et al. 2011). We hypothesize that NMN may enhance mitochondrial oxidative phosphorylation, boost ATP production, improve gut barrier integrity, and support ABCA1‐mediated lipid transport—ultimately promoting the synthesis and secretion of HDL3. This proposed NAD^+^–mitochondria–barrier–HDL3 axis may represent a core mechanism through which NMN alleviates gut–liver axis dysfunction.

Previous studies have demonstrated that NMN supplementation ameliorates various age‐related phenotypes including neurodegenerative disorders, cardiovascular dysfunction, and muscle atrophy (Kiss et al. 2020; Yao et al. 2017; Chandrasekaran et al. 2020). Our earlier work also showed that NMN modulates the gut microbiota and enhances intestinal barrier function in aging mice (Ru et al. 2022). Given that mitochondria are key organelles in energy metabolism and NAD^+^ utilization and are significantly impaired during aging (Klimova et al. 2019; Chen et al. 2020; Lepelley and Crow 2021), it remains to be elucidated whether NMN enhances HDL3 production via improved mitochondrial function, thereby exerting protective effects on the gut–liver axis. Notably, NAD^+^‐sensitive organelles, such as peroxisomes, which contribute to lipid precursor supply for lipoprotein assembly (Hoyland et al. 2024), may also be involved in this regulatory network, though their role in intestinal HDL3 biogenesis during aging is less defined.

This study aims to investigate whether aging impairs HDL3 synthesis through mitochondrial dysfunction, ATP deficiency, and reduced ABCA1 activity, and whether NMN restores HDL3 biosynthesis via the NAD^+^–mitochondria–barrier–HDL3 axis, thereby attenuating age‐related gut–liver dysfunction. Overall, this study seeks to provide novel mechanistic insights and potential therapeutic strategies for age‐related metabolic disorders and liver injury.

## 2 Materials and Methods

### 2.1 Animal Experiments

Animal experiments were carried out following the Regulations and Administration of the Committee of the Institute of Subtropical Agriculture, Chinese Academy of Sciences (No. ISA‐2020‐18). As recommended in the animal welfare protocol, all measures were taken to minimize animal suffering and use only the necessary number of animals required to generate reliable scientific data.

Ten 8‐week‐old male C57BL/6J (Hunan Slake Jingda Experimental Animal Co. Ltd) specific pathogen‐free mice served as the young group (Young), whereas twenty 16‐month‐old male C57BL/6J mice were randomly divided into either the aged group (Aged) or the NMN‐treated aged group (NMN‐Aged), with 10 mice per group. Throughout the experiment, all mice were kept under the same conditions (temperature 25°C ± 2°C, 12‐h light/dark cycle, humidity 60% ± 10%) and had ad libitum access to food and water. Dietary intervention was as follows: mice in the Young and Aged groups were provided with ultrapure water, while mice in the NMN‐Aged group were provided with ultrapure water containing 500 mg/L (w/v) NMN (Ru et al. 2022) (NMN purity ≥ 99.9%; KY‐Rubyberries [Fangchenggang] Biotechnoloies Limited). The body weight, food intake, appearance, and behavioral characteristics of the experimental mice were monitored weekly during the intervention period.

#### 2.1.1 Animal Sample Collection

After 120 days of NMN intervention, all mice were fasted for 12 h and anesthetized by an intraperitoneal injection of pentobarbital sodium (40 mg/kg). Blood samples were collected via orbital puncture, centrifuged at 1000 G for 15 min, and the serum samples were separated and stored at −80°C for subsequent biochemical evaluation. After serum collection, the proximal ileum and liver were immediately separated, rinsed with RNase‐free water, frozen in liquid nitrogen, and stored at −80°C for analysis. The tissues were then fixed in 4% neutral polyformaldehyde and 2.5% glutaraldehyde, respectively, and stored at 4°C for analysis.

#### 2.1.2 Biochemical Analysis

Liver function was evaluated by measuring the levels of aspartate aminotransferase (AST) and alanine aminotransferase (ALT) using commercial assay kits (Solarbio, Beijing, China), according to the manufacturer's instructions. Intestinal barrier function was also assessed by determining the concentrations of diamine oxidase (DAO) and D‐lactic acid (D‐LA) using commercial kits (Solarbio), according to the manufacturer's instructions. The concentration of HDL3 and peroxisomal Acyl‐CoA Oxidase 1 (ACOX1) protein in mice intestinal and intestinal mucosa epithelial (IME) cells was quantified using commercially available enzyme‐linked immunosorbent assay (ELISA) kits, according to the manufacturer's instructions (Jiangsu Enzyme‐free Industry Co. Ltd., Jiangsu, China). Adenosine triphosphate (ATP) concentrations were measured using an ATP detection kit (Beyotime, Shanghai, China), according to the manufacturer's instructions.

#### 2.1.3 Histological Analysis

Histopathological examination was performed as described by Yumeng et al. (Li, Xiao, et al. 2024b). Briefly, a one cubic centimeter sample of ileum and liver tissues was fixed in 4% paraformaldehyde solution for 24 h before dehydrating with different strengths of ethanol. The tissues were then embedded in paraffin, cut into 5‐μm thick sections using a microtome, and stained with hematoxylin and eosin (H&E). The stained sections were finally observed, and the images were captured using CaseViewer software.

#### 2.1.4 Immunohistochemical Analysis

Immunohistochemistry (IHC) staining was performed according to the methods described in our previous study (Li, Liu, et al. 2019). Briefly, 5‐μm‐thick sections were prepared from paraffin‐embedded tissue. The sections were dewaxed, rehydrated, and incubated in 3% H_2_O_2_ for 10 min to eliminate endogenous peroxidase activity. After rinsing with distilled water, the sections were blocked with 10% goat serum for 10 min. The serum was then discarded, the primary antibody working solution was added, and the sections were incubated at 37°C for 2 h (detailed information on primary antibodies is provided in the key resources table). Subsequently, the sections were washed with phosphate‐buffered saline (PBS) and incubated with a biotin‐labeled secondary antibody working solution at 37°C for 30 min. After washing with PBS, the sections were incubated with a horseradish peroxidase‐conjugated streptavidin solution and incubated at 37°C for 20 min. After another wash with PBS, color development was induced by treating the sections with 3,3′‐diaminobenzidine solution for 10 min. Finally, the sections were observed under an inverted microscope. Image pro‐plus 6.0 software (FUJIFILM, USA) was used to measure the staining intensity of each section (Jensen 2013).

#### 2.1.5 Immunofluorescence Analysis

Immunofluorescence (IF) staining was performed according to the methods used in our previous study (Li, Xiao, et al. 2024b). Briefly, isolated ileum tissues were fixed in 4% paraformaldehyde at 4°C for 2 h. Tissues were then embedded in paraffin and cut into 5‐μm sections. The tissue sections were incubated overnight at 4°C with primary antibodies (specific information on primary antibodies is provided in the key resources table). After rinsing with phosphate‐buffered saline, the tissues were incubated with secondary antibodies (diluted 1:200) for 40 min. Immunofluorescence images were acquired using a BZ‐II analyzer (Keyence, Osaka, Japan). Image pro‐plus 6.0 software (FUJIFILM, USA) was used to measure the staining intensity of each section (Jensen 2013).

#### 2.1.6 Telomere Length Analysis

Genomic DNA was extracted from leukocytes, ileum and liver of mice using FastPure Blood DNA Isolation Mini Kit V2 (Vazyme Biotechnology Co. Ltd., Nanjing, China), according to the manufacturer's instructions. The relative telomere length of the samples were measured using quantitative PCR, as previously described (Niu et al. 2021). The primers used were tel1b (F: CGGTTTGTTTGGGTTTGGGTTTGGGTTTGGGTTTGGGTT) and te12b (R: GGCTTGCCTTACCCTTACCCTTACCCTTACCCTTACCCT) to amplify telomeres (T), and primers for the 36B4 gene encoding acid ribosomal phosphoprotein PO (F: ACTGGTCTAGGACCCGAGAAG and R‐TCAATGGTGCCTCTGGAGATT) on mouse chromosome 12 to amplify single‐copy genes (S). All PCRs were performed using a Prism 7700 Sequence Detection System (Applied Biosystems, Foster City, CA, USA), and the thermocycling profiles for both amplicons started with a 10‐min incubation at 95°C to activate AmpliTaq Gold DNA polymerase. For telomere PCR, this was followed by 18 cycles of 15 s at 95°C and 2 min at 54°C. For 36B4 PCR, the profile included 30 cycles of 15 s at 95°C and 1 min at 58°C. Relative telomere length was measured by comparing the ratio of the T repeat copy number to the S copy number, expressed as the ratio of telomere length (T/S).

### 2.2 Cell Experiments

#### 2.2.1 Cell Culture

Mouse normal small intestinal mucosa epithelial (IME) cells and the normal hepatocyte line NCTC1469 were obtained from Fudan Cell Bank (Shanghai, China). The cells were cultured in high‐glucose DMEM (Hyclone, Beijing, China) supplemented with 5% fetal bovine serum (Gibco, Grand Island, USA), 100 U/mL of penicillin, and 100 mg/mL of streptomycin (Macgene, Beijing, China; referred to as complete DMEM) at 37°C in a 5% CO_2_ and 95% air humidified incubator. When the cells grew and fused to 80%–90% confluence, they were collected for further experiments.

IME cells were seeded into 96‐well plates at a density of 1.5 × 10^4^ cells/mL, with 100 μL per well, and cultured at 37°C with 5% CO_2_ and 95% air humidification for 24 h. Next, the media was discarded. In the normal control groups (10 replicates), the medium was replaced with complete DMEM, whereas in the model control groups (10 replicates), the medium was replaced with D‐galactose (200 mM, dissolved in complete DMEM) and cultured for 24 h. After successful establishment of the aging model, the medium was discarded, and the model control group (D‐Gal) (replaced with complete DMEM containing ApoA1 10 μg/mL), ATP intervention group (D‐Gal‐ATP) was replaced with complete DMEM containing ApoA1 10 μg/mL and ATP 50 μM, ATPγS‐AM intervention group (D‐Gal‐ATPγS‐AM) was replaced with complete DMEM containing ApoA1 10 μg/mL and ATPγS‐AM 50 μM, NMN intervention group (D‐Gal‐NMN) was replaced with complete DMEM containing ApoA1 10 μg/mL and NMN 5 μM, the agonist CS‐6253 (MedChem Express, Shanghai, China) intervention group (D‐GAL‐CS‐6253) was replaced with complete DMEM containing ApoA1 10 μg/mL and CS‐6253 1 μM; and the NMN and CS‐6253 synergistic group (D‐Gal‐NMN‐CS‐6253) was replaced with complete DMEM containing ApoA1 10 μg/mL, NMN 5 μM, and CS‐6253 1 μM. All cells were then cultured continuously for another 24 h for subsequent experiments. All cells were then cultured continuously for another 24 h for subsequent experiments.

#### 2.2.2 Co‐Culture of IME and NCTC1469 Cells

IME and NCTC1469 cells were co‐cultured using an embedded cell co‐culture (Transwell Chamber). Specifically, both IME cells and NCTC469 cells were first induced by D‐Gal. Pre‐treated NCTC1469 cells were seeded into the upper chamber at 1 × 10^5^ cells/mL, and pretreated IME cells were seeded into the lower chamber at 1 × 10^5^ cells/mL and supplemented with ApoA1 at 10 μg/mL and NMN at 5 μM. Then the co‐culture system was incubated at 37°C with 5% CO_2_ and 95% air humidified incubators for 24 h, after which subsequent experiments were performed.

#### 2.2.3 Cell Viability Analysis

Cell viability was assessed using the CCK8 kit (Solarbio). Specifically, cells were treated in 96‐well plates, followed by the addition of 10 μL of CCK8 solution per well, and incubated at 37°C for 4 h. Subsequently, absorbance values were measured at 450 nm using a microplate reader (Bio‐Rad Laboratories, Hercules, CA, USA) (Ru et al. 2022).

#### 2.2.4 ApoA1 Overexpression

IME cells were seeded in 6‐well plates at a density of 2.5 × 10^5^ cells per well and cultured overnight until reaching 70%–80% confluence. The experiment included three groups, including a normal control group, a transfection reagent control group (treated with Lipofectamine 2000 only), and an experimental group (transfected with pcDNA3.1(+)‐apoA1 plasmid). For transfection, the experimental group was transfected using Lipofectamine 2000. Specifically, 2.5 μg of plasmid and 5 μL of Lipofectamine 2000 were separately diluted in 125 μL of Opti‐MEM medium, mixed gently, and incubated at room temperature for 20 min to form transfection complexes. Subsequently, 250 μL of the complex was added dropwise to the cells. After 6 h, the medium was replaced with fresh complete medium, and the cells were further cultured for 24–72 h. The overexpression of ApoA1 was validated using quantitative real‐time PCR (qRT‐PCR).

#### 2.2.5 Extracellular Acidification and Oxygen Consumption

Oxygen consumption and extracellular acidification were analyzed by extracellular oxygen consumption assay kit (ABCAM AB197243, Shanghai, China) and glycolysis assay kit (ABCAM AB197244, Shanghai, China), respectively, according to the manufacturer's instructions (Fang et al. 2022; Lang et al. 2022). Fluorescence (Ex 360 nm Em 620 nm for the extracellular acidification assay; Ex 380 nm Em 645 nm for the oxygen consumption assay) was measured every 1.5 min for 2 h by using a microplate reader (Synergy H1; Biotek).

#### 2.2.6 NAD ^+^ and NAD ^+^/NADH Ratio Analysis

The NAD^+^ content and the NAD^+^/NADH ratio in IME cells and the ileum of aging mice were measured using an NAD^+^/NADH assay kit with WST‐8 (Beyotime), according to the manufacturer's instructions.

#### 2.2.7 Determination of Pro‐Inflammatory Cytokines and Lipopolysaccharide

The concentrations of plasma and cell culture medium pro‐inflammatory cytokines, including tumor necrosis factor‐α (TNF‐α), interleukin‐1β (IL‐1β), and interleukin‐6 (IL‐6), and the concentrations of liver and Intestinal LPS were detected using commercially available ELISA kits (Jiangsu Enzyme‐free Industry), according to the manufacturer's instructions.

#### 2.2.8 Transmission Electron Microscopy Analysis

Transmission electron microscopy (TEM) was used to visualize the ultrastructural characteristics of the IME cells, NCTC1469 cells, ileum, and liver. The samples were collected and first fixed with a solution containing 2% paraformaldehyde and 2.5% glutaraldehyde at 4°C for 1 h and then fixed in 1% osmium tetroxide for 1 h. The tissues were dehydrated in a graded ethanol series before being infiltrated with epoxy resin. Ultrathin sections (50 nm) were prepared and stained using 0.2% lead citrate and 4% uranyl acetate. Finally, the sections were observed under an HT7700 electron microscope.

#### 2.2.9 Cholesterol Efflux Assay

IME cells were cultured with fluorescent‐labeled cholesterol reagent (ABCAM AB196985, Shanghai, China) for 24 h and then treated with NMN or combined with ATP for 24 h and serum‐free medium containing ApoA‐1 or HDL for another 4 h. Finally, the fluorescence intensity of cell culture supernatant and intracellular lipid content was determined by multifunctional microplate reader (Ex/Em = 485/523 nm). The cholesterol efflux rate was equal to the fluorescence intensity of cell culture supernatant divided by the sum of the fluorescence intensity of cell culture supernatant and intracellular lipid content.

#### 2.2.10 Peroxisomal ACOX1 Enzymatic Activity Assay

ACOX1 enzymatic activity was assessed by measuring H₂O₂ production from very‐long‐chain acyl‐CoA substrates (Lu et al. 2024). In brief, ileum tissue homogenates (prepared in 20 mM potassium phosphate buffer, pH 7.4, containing 1 mM EDTA and 0.1% Triton X‐100) were incubated at 37°C in a reaction mixture containing 50 μM Amplex Red (Beyotime Biotechnology, Shanghai, China), 0.2 U/mL horseradish peroxidase (HRP; Solarbio Science & Technology, Beijing, China), and 50 μM palmitoyl‐CoA or C26:0‐CoA (MedChem Express, Shanghai, China) as substrate. Fluorescence (Ex/Em = 560/590 nm) was monitored every 2 min for 60 min using a Synergy H1 microplate reader (BioTek, Winooski, VT, USA). Enzyme activity was calculated based on a standard curve of H₂O₂ and normalized to total protein content (nmol H₂O₂/min/mg protein). Reactions without substrate or with heat‐inactivated samples served as negative controls.

#### 2.2.11 Quantitative Reverse Transcription PCR (RT‐qPCR) Analysis

The RT‐qPCR method was used to determine the gene expression of IME cells, NCTC1469 cells, ileum, and liver tissues, according to a previous method (Li, Sun, et al. 2024a). Total RNA was extracted from the samples using Trizol reagent. cDNA was synthesized from the total RNA by reverse transcription using an RT‐qPCR kit (TaKaRa Biomedical Technology, Beijing, China). The primers used in the study were designed and synthesized by Sangon Biotech (Shanghai, China). The RT‐qPCR was performed on a CFX‐96 real‐time platform (Bio‐Rad, Hercules, CA, USA) using Hieff UNICON Universal Blue qPCR SYBR Master Mix kits (Yeasen, Shanghai, China). The thermal cycling conditions were as follows: an initial denaturation at 95°C for 30 s, followed by 40 cycles at 95°C for 5 s and 58°C for 5 s, with a final extension step at 72°C for 30 min. The relative expression levels were determined using the $2^{-∆∆C_{t}}$ method, with β‐actin as an endogenous control. The primer details are provided in Table S1.

#### 2.2.12 Western Blotting (WB) Analysis

Ileum tissues were lysed with RIPA buffer containing 0.1 mM Na_3_VO_4_, 1 mM NaF, 1 mM 4‐(2‐aminoethyl)‐benzenesulfonyl fluoride hydrochloride (AEBSF), and 5 mg/mL aprotinin (Sigma‐Aldrich). Total proteins were quantified using the BCA (bicinchoninic acid) assay kit and Bradford assay (Thermo Fisher, 23227). Protein samples were loaded on SDS‐PAGE and transferred to polyvinylidene fluoride (PVDF) membranes. The membranes were blocked for 1 h at RT with 5% skim milk. After washing with TBST, primary antibodies OXPHOS (ABclonal, Wuhan, China) (1:1000) were used. After washing with TBST, membranes were incubated with horseradish peroxidase (HRP)‐conjugated secondary antibodies (1:10,000) for 1 h at RT. The image was detected using the ChemiDoc (Thermo Fisher, iBrightCL1500).

### 2.3 Statistical Analyses

Statistical differences between groups were analyzed using the Student's *t*‐test. One‐way analysis of variance (ANOVA) followed by Dunnett's multiple comparison tests was used to determine whether there were significant differences between more than two treatments. Statistical significance was indicated as follows: ns, no significance, * for *p* < 0.05, and ** for *p* < 0.01, with both levels considered statistically significant. The sample size and type of statistical tests used are described in the legend of each figure.

## 3 Results

### 3.1 Aging Impairs Intestinal Function and Reduces Intestinal‐Derived HDL3 Levels

**FIGURE 1:**
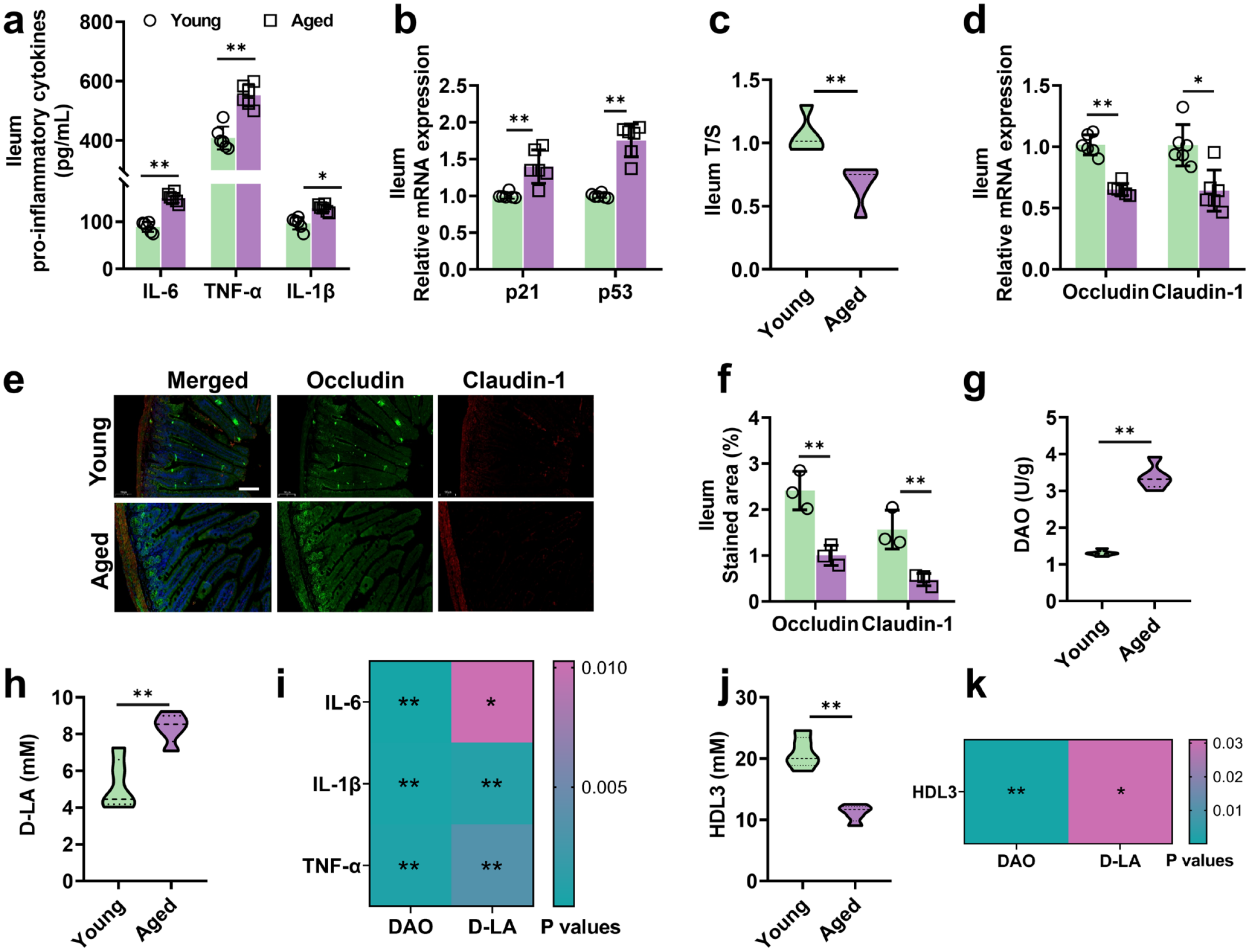
Aging impairs intestinal function and reduces intestinal‐derived HDL3 levels. (a) Ileum IL‐6, TNF‐α and IL‐1β levels, *n* = 5; (b) relative mRNA expression of *p21* and *p53*, *n* = 5; (c) relative telomere length (T/S), *n* = 5; (d) relative mRNA expression of *Occludin* and *Claudin‐1*, *n* = 6; (e, f) representative images (scale bar: 100 μm) and quantitative analysis of Occludin and Claudin‐1 measured by IF staining, *n* = 6 images from *n* = 3 independent experiments; (g, h) serum DAO and D‐LA levels, *n* = 5; (i) correlation analysis of SASP and intestinal barrier function indicators; (j) intestinal‐derived HDL3 levels, *n* = 5; and (k) correlation between intestinal‐derived HDL3 levels and intestinal barrier function indicators. Data are express as the mean ± SEM. *p < 0.05, **p < 0.01. DAO, diamine oxidase; D‐LA, D‐lactic acid; IF, immunofluorescence; SASP, senescence‐associated secretory phenotype.

Aging impairs the integrity of the intestinal barrier and reduces the synthesis efficiency of intestinal‐derived bioactive substances (Zhang et al. 2025). Through comparative analysis of young and aged mouse models, this study found that the ileum of aged mice exhibited typical senescence‐associated features, including significant upregulation of pro‐inflammatory cytokines (IL‐6, IL‐1β, and TNF‐α) as part of the senescence‐associated secretory phenotype (SASP) (Figure 1a), marked telomere shortening (Figure 1b), and elevated mRNA expression of cell cycle regulators *p53* and *p21* (Figure 1c). Assessment of intestinal barrier function revealed a significant decrease in tight junction proteins including Occludin and Claudin‐1 (Figure 1d–f) alongside increased plasma levels of intestinal barrier damage markers DAO and D‐LA (Figure 1g,h) in Aged group. Correlation analysis confirmed a strong positive association between intestinal dysfunction and aging biomarkers (Figure 1i). Notably, HDL3—the most antioxidative and anti‐inflammatory subtype of Intestinal‐derived high‐density lipoprotein—was significantly reduced in the ileum of aged mice (Figure 1j). Further analysis demonstrated a significant negative correlation between intestinal barrier impairment and HDL3 levels (Figure 1k), indicating that age‐related intestinal barrier dysfunction is closely linked to the decline of Intestinal‐derived HDL3.

### 3.2 Mitochondrial Dysfunction‐Mediated ATP Deficiency Suppresses HDL3 Synthesis in Aging Intestinal Cells

**FIGURE 2:**
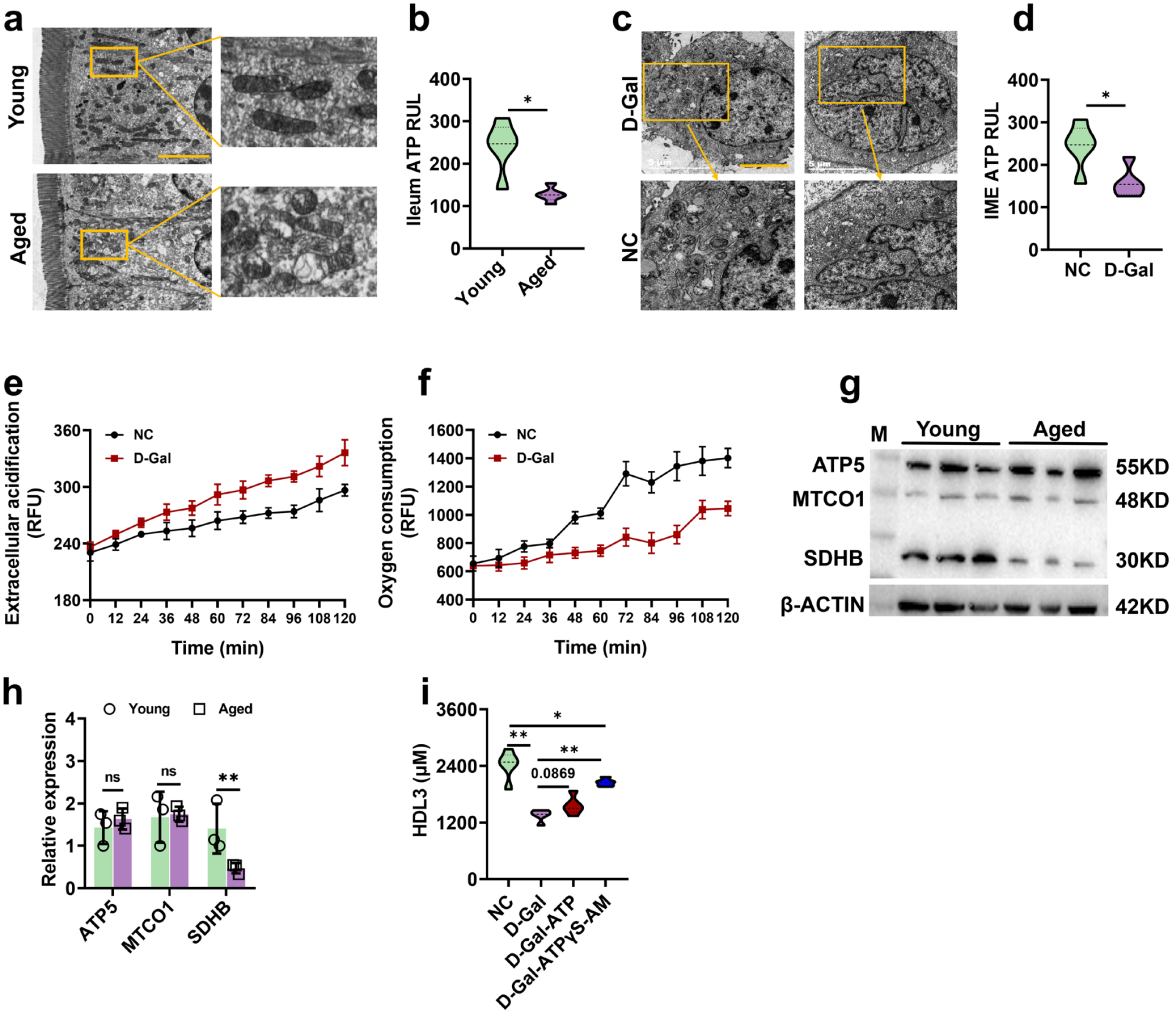
Mitochondrial dysfunction‐mediated ATP deficiency suppresses HDL3 synthesis in aging intestinal cells. (a) Representative images (scale bar: 5 μm) of intestinal cell microstructure measured by TEM, *n* = 18 images from *n* = 3 independent experiments; (b) ileum ATP levels, *n* = 5; (c) representative images (scale bar: 5 μm) of IME microstructure measured by TEM, *n* = 18 images from *n* = 3 independent experiments; (d) IME ATP levels, *n* = 5; (e) glycolysis assay measured as cytoplasmic acidification, the fluorescence signal was enhanced with the increase of acidification degree, *n* = 4; (f) oxygen consumption, as mitochondrial respiration depletes the oxygen within the assay medium, quenching of the fluorescent dye is reduced, and the fluorescence signal increases proportionately, *n* = 4; (g, h) OXPHOS protein expression levels in the ileum, *n* = 3; and (i) exogenous ATPγS‐AM (50 μM) partially restored HDL3 synthesis in senescent IME cells, whereas native ATP (50 μM) had no significant effect, *n* = 5. Data are express as the mean ± SEM. *p < 0.05, **p < 0.01. D‐Gal: D‐galactose; NC, normal control.

Adequate ATP supply is essential for HDL3 biosynthesis. Here, we first examined the function of mitochondria, the primary ATP producers. TEM revealed that mitochondria in the ileum of aged mice exhibited swelling, deformation, and vacuolation compared to those in young mice (Figure 2a). Consistently, ATP levels were significantly lower in aged ileum tissues (Figure 2b). In cellular experiments, we established an IME senescence model using D‐Gal and observed similar mitochondrial damage and reduced ATP levels (Figure 2c,d). ECAR and OCR assays demonstrated that senescent IME cells exhibited significantly higher acidification (Figure 2e) and markedly reduced oxygen consumption (Figure 2f) compared to controls. Furthermore, the expression levels of key OXPHOS proteins were decreased in aged ileum (Figure 2g–i).

To determine whether the decline in *intracellular* ATP due to mitochondrial dysfunction directly limits HDL3 production, we treated senescent IME cells with a membrane‐permeable ATP analog (ATPγS‐AM, 50 μM) to specifically elevate cytosolic ATP levels. As shown in Figure 2j, ATPγS‐AM treatment significantly restored HDL3 secretion. In contrast, supplementation with native ATP (50 μM), which cannot cross the plasma membrane, failed to rescue HDL3 production, confirming that the effect is dependent on intracellular ATP availability rather than extracellular purinergic signaling. These results demonstrate that mitochondrial dysfunction–driven ATP depletion in aging intestinal epithelial cells directly impairs ABCA1‐mediated HDL3 biogenesis. The partial restoration of HDL3 by ATPγS‐AM also suggests that, beyond energy supply, age‐related alterations in the expression or activity of key biosynthetic components may further constrain HDL3 output. In addition to mitochondrial dysfunction, aging was also associated with impaired peroxisomal metabolism, reflected by reduced ACOX1 expression and activity (Figure S1a,b).

### 3.3 ABCA1 Downregulation Limits HDL3 Synthesis in Aging

**FIGURE 3:**
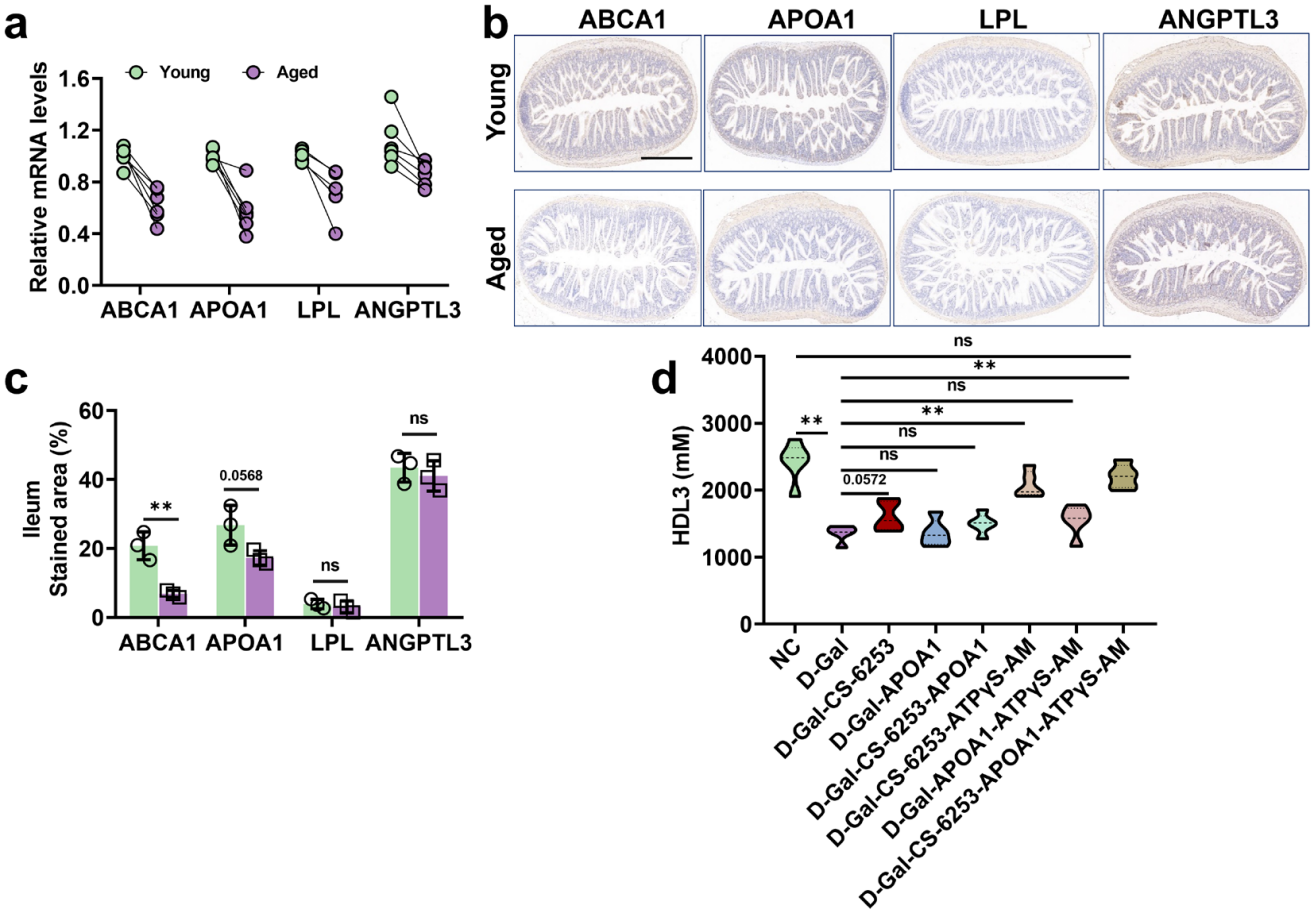
ABCA1 downregulation limits HDL3 synthesis in aging. (a) Relative mRNA expression of *ABCA1*, *ApoA1*, *LPL*, and *ANGPTL3* in ileum, *n* = 5; (b, c) representative images (scale bar: 50 μm) and quantitative analysis of ABCA1, ApoA1, LPL, and ANGPTL3, measured by IHC staining, *n* = 3; and (d) activation of ABCA1 expression combined with ATPγS‐AM supplementation enhances cellular HDL3 synthesis capacity *n* = 5. Data are express as the mean ± SEM. **p < 0.01. ABCA1, ATP‐binding cassette transporter 1; ANGPTL3, angiopoietin‐like3; CS‐6253, ABCA1 activators; D‐Gal, D‐galactose; HDL3, high‐density lipoprotein 3; IHC, immunohistochemistry; IME, intestinal mucosa epithelial; LPL, lipoprotein lipase; NC, normal control.

To identify the key targets limiting HDL3 synthesis during aging, we examined the mRNA and protein expression levels of critical HDL3‐associated proteins including ABCA1, ApoA1, LPL, and ANGPTL3 in intestinal tissues. The results demonstrated significantly lower mRNA levels of *ABCA1*, *ApoA1*, *LPL*, and *ANGPTL3* in aged mice compared to the young group (Figure 3a). At the protein level, both ABCA1 and ApoA1 showed significantly decreased expression in aged mice, whereas LPL and ANGPTL3 protein levels remained unchanged compared to the young group (Figure 3b,c). Furthermore, we overexpressed ApoA1, activated ABCA1 expression using the agonist CS‐6253, or supplemented ATPγS‐AM in senescent IME cells. Notably, HDL3 synthesis was significantly restored only when ATPγS‐AM supplementation was combined with ABCA1 activation (Figure 3d), suggesting that mitochondrial oxidative phosphorylation capacity, along with ABCA1, plays a pivotal role in mediating intestinal HDL3 synthesis.

### 3.4 Aging Impairs ABCA1‐Mediated Cholesterol Efflux and Reduces HDL3 Synthesis

**FIGURE 4:**
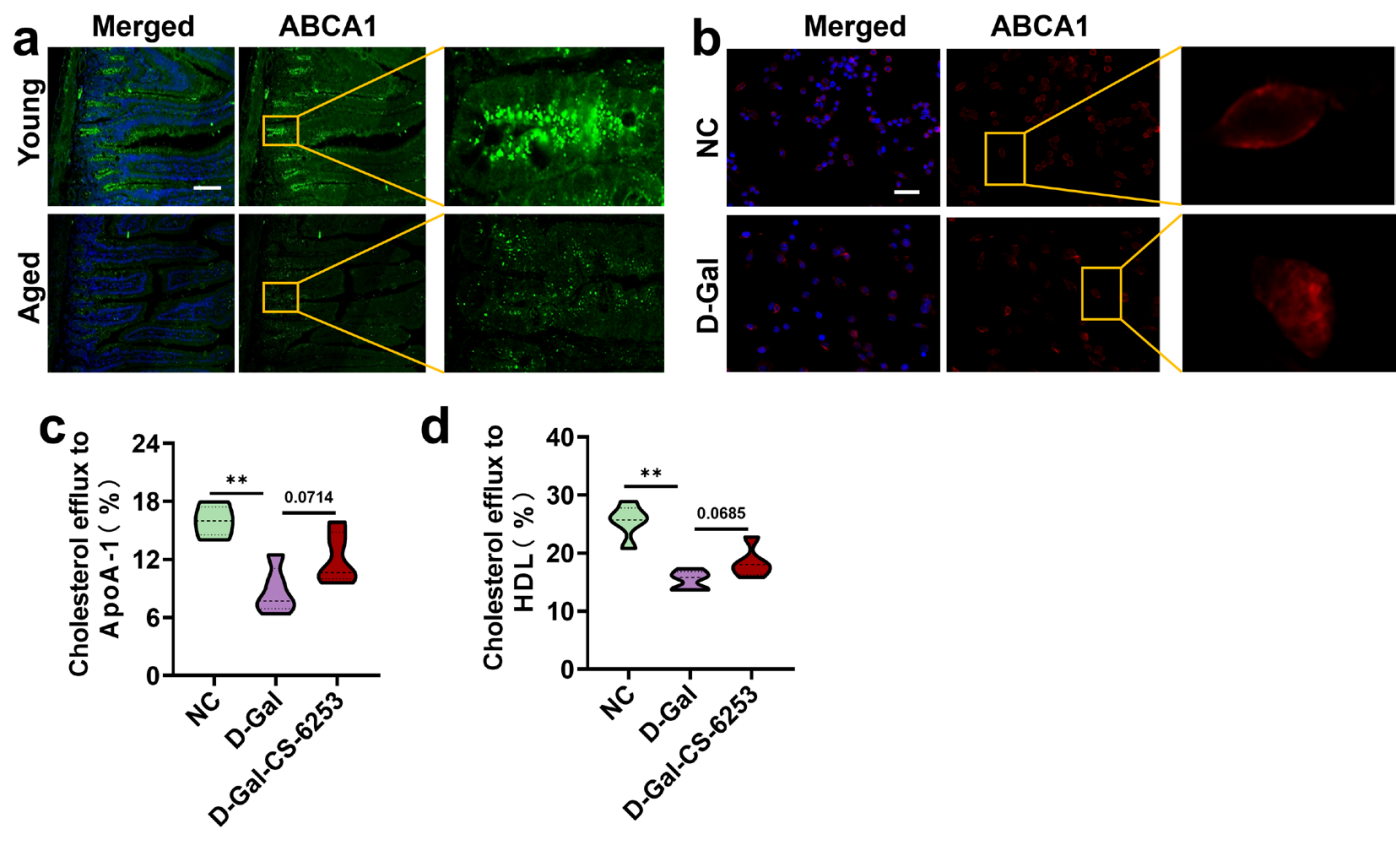
Aging impairs ABCA1‐mediated cholesterol efflux and reduces HDL3 synthesis. (a, b) Representative images (scale bar: 100 μm) of ABCA1 measured by IF staining in ileum and IME, *n* = 6 images from *n* = 3 independent experiments; and (c, d) efficiency of cholesterol efflux to ApoA‐1 and HDL, *n* = 5. Data are expressed as the mean ± SEM. **p < 0.01. ABCA1, ATP‐binding cassette transporter 1; CS‐6253, ABCA1 activators; D‐Gal, D‐galactose; IF, immunofluorescence; IME, intestinal mucosa epithelial; NC, normal control.

The efficiency of ABCA1 localization on the cell membrane determines the synthesis capacity of HDL3. This study found a significant reduction in ABCA1 membrane localization on intestinal membranes of aging mice, a conclusion that was similarly validated in cellular models (Figure 4a,b). Cholesterol efflux assays demonstrated a significant reduction in the efflux of cholesterol to both ApoA‐1 and HDL in senescent cells, whereas activation of ABCA1 expression partially restored cholesterol efflux in these cells (Figure 4c,d). These results are consistent with the conclusion that effective enhancement of intestinal HDL3 synthesis in aging cells can only be achieved when ABCA1 expression is activated and exogenous ATP is supplemented. This suggests that targeting the activation of ABCA1 expression to enhance its membrane localization, in combination with intracellular ATP supply, is an effective strategy to improve HDL3 synthesis capacity in aging cells.

### 3.5 NMN Modulates Mitochondrial Function to Boost ATP Production in the Aging Intestine

**FIGURE 5:**
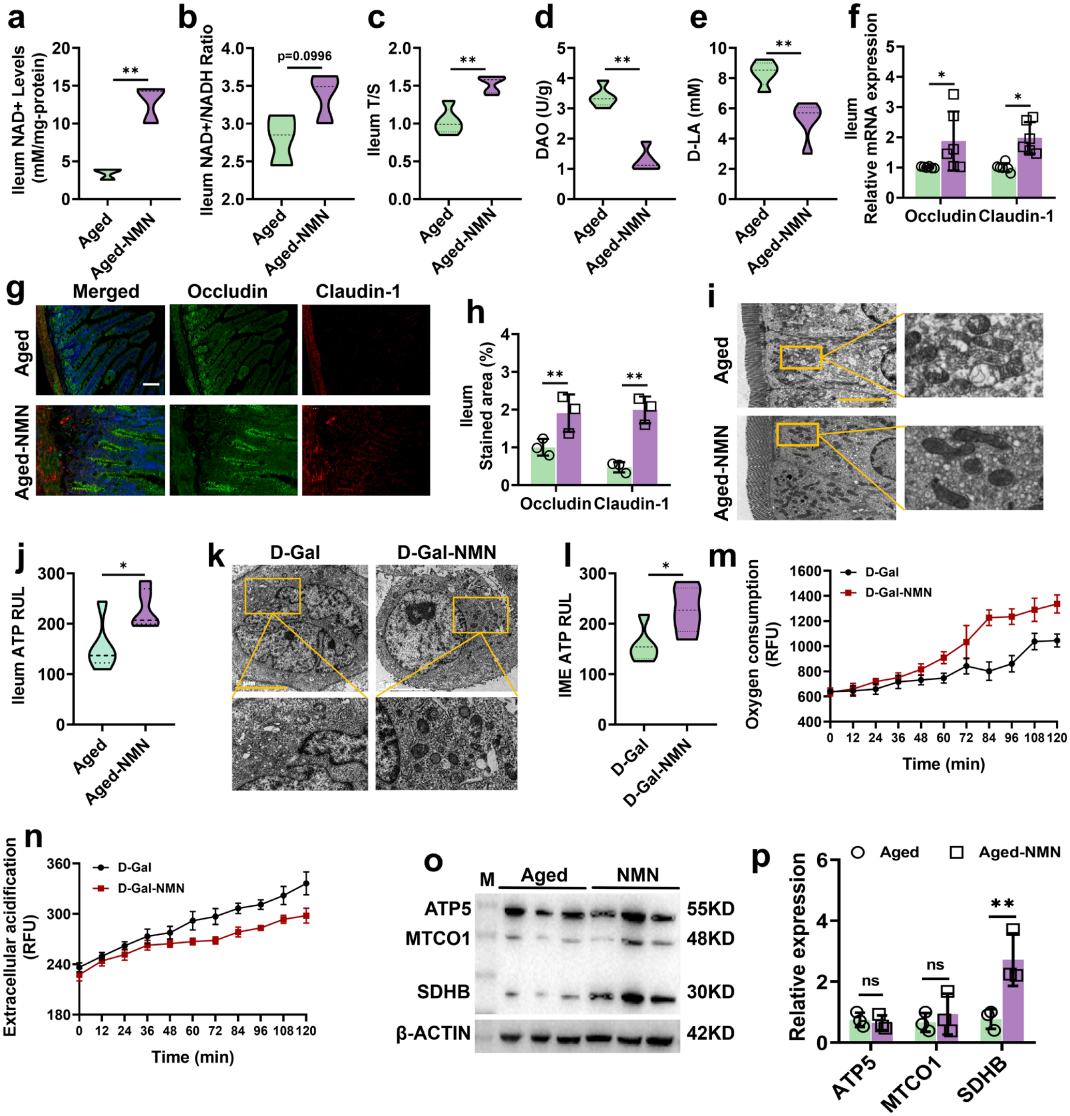
NMN modulates mitochondrial function to boost ATP production in the aging intestine. (a, b) NADH levels and NAD^+^/NADH ratio in ileum, *n* = 3; (c) relative telomere length in ileum (T/S), *n* = 5; (d, e) DAO and D‐LA levels in serum, *n* = 5; (f) ileum relative mRNA expression of *Occludin* and *Claudin‐1*, *n* = 6; (g, h) representative images (scale bar: 100 μm) and quantitative analysis of Occludin and Claudin‐1 measured by IF staining, *n* = 6 images from *n* = 3 independent experiments; (i, k) representative images (scale bar: 5 μm, scale bar: 1 μm) of ileum and IME cell structure measured by TEM, *n* = 18 images from *n* = 3 independent experiments; (j, l) ATP levels in ileum and IME cell, *n* = 5; (m) glycolysis assay measured as cytoplasmic acidification, the fluorescence signal was enhanced with the increase of acidification degree, *n* = 4; (n) oxygen consumption, as mitochondrial respiration depletes the oxygen within the assay medium, quenching of the fluorescent dye is reduced, and the fluorescence signal increases proportionately, *n* = 4; and (o, p) OXPHOS protein expression levels in the ileum, *n* = 3. Data are express as the mean ± SEM. *p < 0.05, **p < 0.01. DAO, diamine oxidase; D‐Gal, D‐galactose; D‐LA, D‐lactic acid; IF, immunofluorescence; IME, intestinal mucosa epithelial; NC, normal control.

Here, we further investigated whether exogenous NMN supplementation could delay intestinal cell senescence and improve mitochondrial function to enhance ATP production. Our results demonstrated that NMN administration significantly increased NAD^+^ levels and the NAD^+^/NADH ratio in intestinal tissues of aged mice, while also markedly elongating relative telomere length (T/S ratio) (Figure 5a–c). Intestine functional analyses revealed that NMN lowered serum levels of DAO and D‐LA in aged mice (Figure 5d,e), and upregulated both mRNA and protein expression of tight junction proteins Occludin and Claudin‐1 in intestinal tissues (Figure 5f–h). Furthermore, TEM revealed that NMN ameliorated age‐related mitochondrial swelling, deformation, and vacuolization in the ileum (Figure 5i). Notably, NMN supplementation significantly increased ATP levels in aged ileum (Figure 5j). In vitro, NMN also alleviated mitochondrial damage and restored ATP production in senescence‐induced intestinal epithelial cells (Figure 5k,l). ECAR and OCR assays showed that NMN significantly reduced glycolytic acidification (Figure 5m) and enhanced mitochondrial respiration (Figure 5n) in aged IME cells compared to controls. In addition, expression of key OXPHOS proteins was restored in aged ileum following NMN treatment (Figure 5o,p). Collectively, these findings suggest that NMN enhances ATP biosynthesis in aging intestinal tissues by promoting mitochondrial energy metabolism. Notably, NMN not only restored mitochondrial function but also preserved peroxisomal integrity in the aging intestine, as evidenced by maintained ACOX1 protein levels and enzymatic activity (Figure S1a,b).

### 3.6 NMN Enhances Intestinal HDL3 Synthesis in the Aging Intestine

**FIGURE 6:**
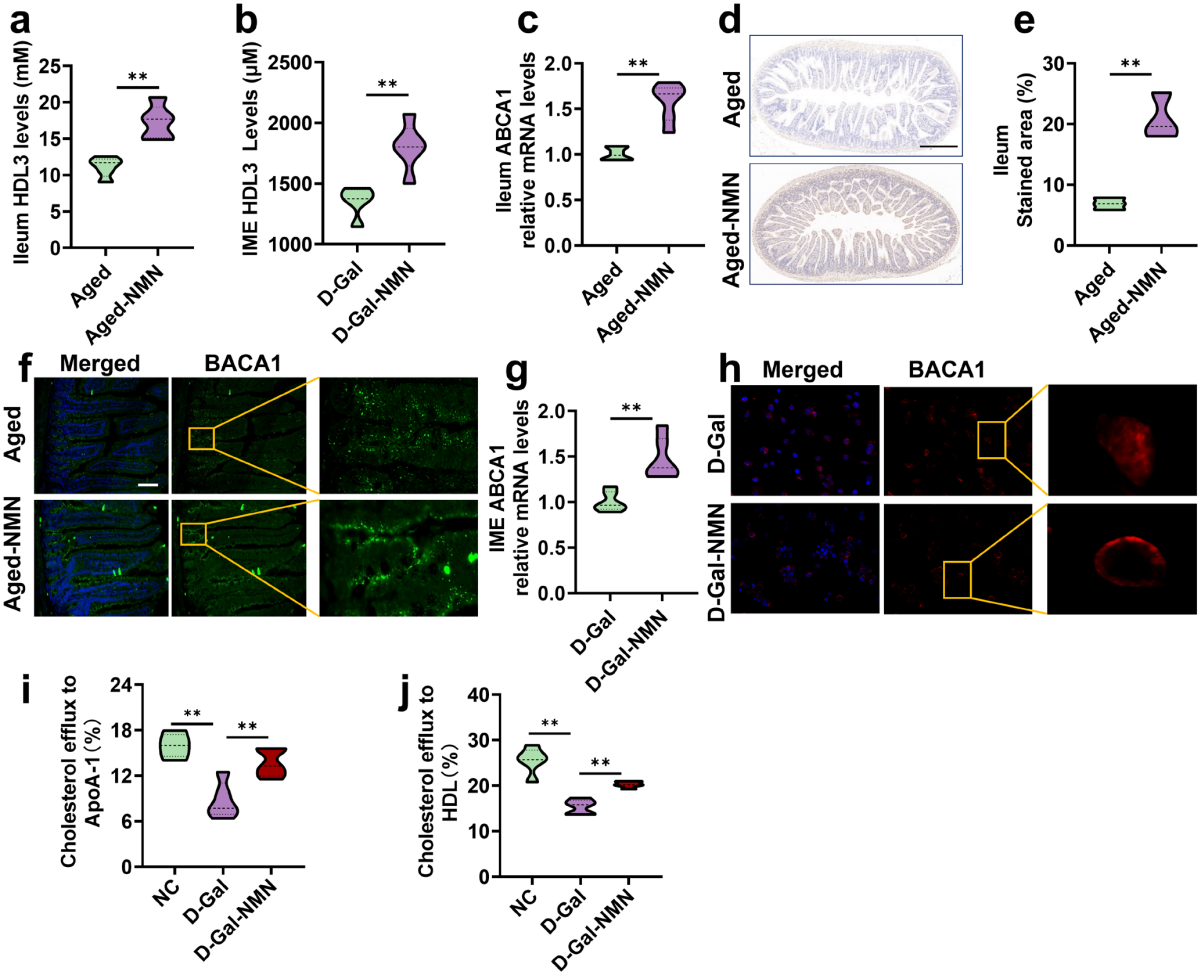
NMN enhances intestinal HDL3 synthesis in the aging intestine. (a, b) NMN enhanced HDL3 synthesis capacity in the ileum and IME cells, *n* = 5; (c–e) NMN increased the relative expression of ABCA1 mRNA and protein in the ileum. *n* = 3; (f, h) representative images (scale bar: 100 μm) of ABCA1 localization to the cell membrane measured by IF staining, *n* = 6 images from *n* = 3 independent experiments; (g) NMN increased the relative expression of ABCA1 mRNA in the IME cells. *n* = 3; and (i, j) NMN enhanced cholesterol efflux to ApoA‐1 and HDL in aging cells, *n* = 5. Data are expressed as the mean ± SEM. **p < 0.01. ABCA1, ATP‐binding cassette transporter 1; D‐Gal, D‐galactose; HDL, high‐density lipoprotein; IF, immunofluorescence; IME, intestinal mucosa epithelial; NC, normal control.

To investigate the role of NMN in regulating age‐related intestinal HDL3 synthesis, we assessed changes in HDL3 levels in the intestinal tissues of aged mice following NMN intervention. The results showed that NMN significantly increased intestinal HDL3 levels in aged mice (Figure 6a), and a similar trend was observed in D‐Gal‐treated IME cells (Figure 6b). Further analysis revealed that NMN upregulated the relative expression of key genes and proteins involved in HDL3 synthesis, particularly ABCA1, in the intestinal tissues and cells of aged mice (Figure 6c,d,g). This enhanced expression promoted ABCA1 localization to the cell membrane, facilitating its reverse cholesterol transport function (Figure 6f,h). These findings were further confirmed by cholesterol and phospholipid efflux assays, which demonstrated that NMN significantly enhanced the efflux capacity of cholesterol and phospholipids in aging cells (Figure 6i,j), thereby providing additional lipid substrates for the biosynthesis of intestinal‐derived HDL3.

### 3.7 NMN Attenuates Age‐Related Liver Injury via Intestinal‐Derived HDL3

**FIGURE 7:**
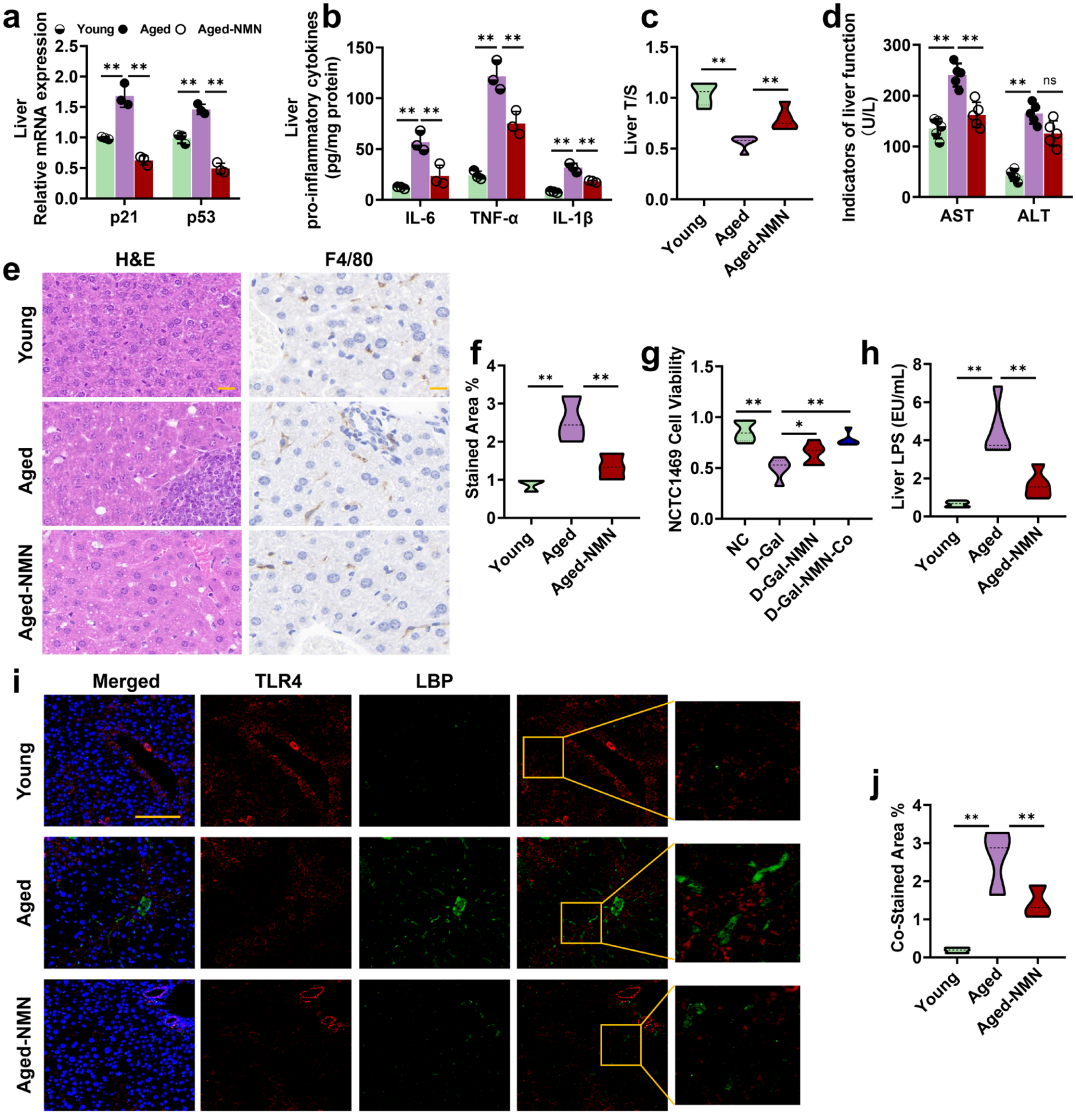
NMN attenuates age‐related liver injury via intestinal‐derived HDL3. (a) Relative mRNA expression of *p21* and *p53* in liver, *n* = 5; (b) liver IL‐6, TNF‐α and IL‐1β levels, *n* = 5; (c) relative telomere length in liver (T/S), *n* = 5; (d) AST and ALT levels in serum, *n* = 5; (e) representative images (scale bar: 50 μm) of H&E and IHC staining of liver, *n* = 18 images from *n* = 3 independent experiments; (f) quantitative analysis of F4/80 measured by IHC staining *n* = 18 images from *n* = 3 independent experiments; (g) cell viability of NCTC146 co‐cultured with NMN or IME cells, *n* = 5; (h) LPS levels in liver, *n* = 5; and (i, j) representative images (scale bar: 100 μm) and quantitative analysis of TLR4 and LBP co‐stain measured by IF staining, *n* = 6 images from *n* = 3 independent experiments. Data are express as the mean ± SEM. *p < 0.05, **p < 0.01. ALT, alanine aminotransferase; AST, aspartate aminotransferase; Co, co‐culture; D‐Gal: D‐galactose; IF, immunofluorescence; NC, normal control.

Intestinal‐derived HDL3 has been shown to prevent various types of liver inflammatory damage (Han, Onufer, et al. 2021). This study explores whether NMN‐mediated intestinal‐derived HDL3 can alleviate age‐related liver injury. The results demonstrated that NMN treatment effectively improves the hepatic aging phenotype in aged mice. Specifically, NMN intervention significantly inhibited the expression of age‐related genes *p21* and *p53*, as well as the levels of SASP in the liver (Figure 7a,b). It also extended telomere length (Figure 7c) and reduced serum liver function markers ALT and AST (Figure 7d). Histological analysis using H&E staining and F4/80 showed that NMN treatment significantly decreased hepatic inflammation, fibrosis, and lipid deposition in aged mice (Figure 7e,f).

In cellular experiments, co‐culture of IME and NCTC1469 cells confirmed that NMN alleviated D‐Gal‐induced cellular senescence in NCTC1469 cells, with enhanced effects when co‐cultured with IME cells (Figure 7g), suggesting a crucial role of gut‐derived HDL3 in improving liver inflammatory injury. Mechanistic analysis revealed a significant reduction in LPS levels in the livers of NMN‐treated aged mice (Figure 7h). Immunofluorescence co‐localization analysis showed that NMN notably inhibited the interaction between TLR4 and LPS‐binding protein (LBP) in the liver (Figure 7i,j), consistent with the hypothesis that gut‐derived HDL3 occupies TLR4 binding sites, reducing LPS‐induced hepatic inflammatory damage.

## 4 Discussion

Aging is a complex biological process characterized by a progressive decline in multiple physiological functions, which is particularly pronounced in highly metabolic tissues such as the intestine and liver (Loomba et al. 2017; Li et al. 2024; Yang et al. 2024). Extensive studies have confirmed that the age‐related reduction in intracellular nicotinamide adenine dinucleotide (NAD^+^) levels represents a key factor exacerbating the aging process (Jiao et al. 2022; Li et al. 2023). As an essential coenzyme involved in energy metabolism and cellular homeostasis, the decline in NAD^+^ impairs mitochondrial oxidative phosphorylation, reduces ATP production, and consequently leads to systemic metabolic dysregulation (Gulyamova et al. 2001; Yoshino et al. 2018). In recent years, the gut–liver axis has been recognized as a critical interface for maintaining metabolic balance and immune homeostasis, and its dysfunction is closely associated with various age‐related diseases including non‐alcoholic fatty liver disease, insulin resistance, and systemic inflammation (Li et al. 2025; Loguercio et al. 2002). Of particular note, aging is often accompanied by compromised intestinal barrier integrity and translocation of microbial metabolites such as LPS into the portal circulation, thereby activating hepatic inflammatory pathways (Babu and Mohanty 2023; Jiang et al. 2021). However, the mechanisms through which aging affects the biosynthesis of gut‐specific anti‐inflammatory factors, particularly intestinal‐derived HDL3, and its regulatory role in gut–liver crosstalk remain poorly understood.

HDL3, synthesized and secreted by intestinal epithelial cells, exhibits potent anti‐inflammatory and antioxidant activities. It effectively neutralizes LPS and mitigates liver inflammatory injury (Chen and Wang 2021; Wang et al. 2022). Although lipid metabolic disorders in aging have been linked to impaired ABCA1‐mediated cholesterol efflux (Han, Onufer, et al. 2021), it remains unclear whether aging directly compromises the synthetic capacity of intestinal HDL3 and whether this process involves dysfunction of the NAD^+^–mitochondria–metabolism axis. Therefore, elucidating the alterations and regulatory mechanisms of intestinal HDL3 biosynthesis in the context of aging, thereby establishing a novel mechanistic link between impaired intestinal metabolism and age‐related hepatic pathology, is not only of theoretical significance but may also provide new interventional strategies for delaying aging‐related liver diseases.

In this study, we observed a significant decline in the synthetic capacity for intestinal‐derived HDL3 in the intestinal tissue of aged mice. HDL3 biogenesis is an energy‐intensive process that relies on efficient lipid transport machinery and apolipoprotein assembly systems (Loguercio et al. 2002; Duka et al. 2013). Our results demonstrate that aging induces severe ultrastructural abnormalities and functional impairment of mitochondria in intestinal epithelial cells, leading to insufficient ATP biosynthesis. This energy deficiency critically constrains the ability of enterocytes to synthesize HDL3, which is consistent with previous reports linking mitochondrial dysfunction to impaired cellular lipid metabolism (Chen and Wang 2021; Knez et al. 2017; Wang et al. 2024; Lin et al. 2024). More importantly, this study revealed that both the expression and, particularly, the membrane localization of ABCA1, a key transporter responsible for phospholipid and cholesterol efflux, are substantially compromised in aged intestinal cells. The functional relevance of these observations was confirmed through rescue experiments, in which only the combined administration of exogenous ATPγS‐AM and enforced expression of ABCA1, but neither intervention alone, fully restored HDL3 biosynthesis. These findings indicate that both energetic substrates and functional transporter availability constitute non‐redundant limiting factors in age‐related HDL3 deficiency, thereby providing mechanistic insights that extend beyond previous studies focused primarily on the transcriptional regulation of HDL‐associated genes.

Peroxisomes, organelles highly sensitive to cellular NAD^+^ fluctuations, may also play a pivotal, albeit indirect, role in HDL3 biogenesis. Unlike mitochondria, which can retain their NAD^+^ pools under systemic NAD^+^ depletion (Hoyland et al. 2024), peroxisomes are particularly vulnerable to age‐related NAD^+^ decline. Given their essential functions in ether phospholipid synthesis and very‐long‐chain fatty acid β‐oxidation (Wang et al. 2024), peroxisomal dysfunction could compromise the availability of key lipid precursors required for HDL3 assembly and impair ABCA1 stability at the plasma membrane. In support of this notion, we found that both ACOX1 protein levels and its enzymatic activity were significantly reduced in the ileum of aged mice, and NMN supplementation effectively preserved peroxisomal integrity. These findings suggest that, in addition to mitochondrial ATP deficiency, age‐related peroxisomal impairment may constitute a parallel mechanism contributing to intestinal HDL3 deficiency.

Given the established role of NAD^+^ depletion in age‐related mitochondrial dysfunction (Tarantini et al. 2019; Kiss et al. 2020; Miao et al. 2020), it was hypothesized that NMN supplementation might represent a viable therapeutic strategy to counteract the observed HDL3 deficiency. This study demonstrated that NMN administration effectively elevates NAD^+^ bioavailability, rescues oxidative phosphorylation capacity, and enhances ATP generation. These observations align with previous reports demonstrating that NAD^+^ precursors improve mitochondrial function across various aging tissues (Okabe et al. 2022; Loreto et al. 2023). Notably, NMN‐induced enhancement of mitochondrial energy metabolism was accompanied by significant augmentation of ABCA1 expression and membrane localization. This suggests a previously unrecognized role for NAD^+^‐dependent energy metabolism in regulating the trafficking and stability of this critical lipid transporter, potentially through SIRT1‐mediated deacylation pathways known to modulate ABCA1 activity (Kiss et al. 2020; Tian et al. 2024). Consequently, NMN treatment enhanced cholesterol efflux capacity and provided essential lipid substrates and catalytic machinery for HDL3 assembly, ultimately increasing its production. Although the current study establishes NMN as an effective NAD^+^ precursor in this context, we acknowledge that a direct comparative assessment with other NAD^+^‐boosting agents, such as nicotinamide (NAM), was not performed. Future studies incorporating NAM as a control would help delineate whether the benefits observed here are specific to NMN or represent a common effect of NAD^+^ repletion, thereby further clarifying the relative therapeutic potential of different NAD^+^ precursors in aging‐related intestinal and hepatic dysfunction.

Building upon these findings regarding the role of NMN in restoring intestinal derived HDL3 synthesis, we further investigated its functional impact on systemic homeostasis, particularly liver health (Nakajo et al. 2023; de Picciotto et al. 2016). Phenotypically, the reduction in hepatic *p*21 and *p53* expression following NMN treatment further suggests that restoring intestinal HDL3 production may attenuate cellular senescence programs in the liver. Moreover, the functional significance of rebuilt intestinal HDL3 biosynthesis was corroborated by a marked reduction in hepatic LPS levels and suppression of the TLR4/LBP inflammatory axis in NMN‐treated aged mice. This aligns with the established role of HDL3 in binding and neutralizing LPS, thereby preventing its interaction with Toll‐like receptors and subsequent activation of inflammatory cascades (Chen and Wang 2021; Gautam et al. 2025). Furthermore, co‐culture experiments provided direct causal evidence that NMN‐treated IME cells attenuated senescence and injury in hepatocytes, an effect dependent on the production of functional HDL3.

In conclusion, this study delineates a comprehensive mechanistic pathway wherein aging compromises intestinal HDL3 biosynthesis through concurrent mitochondrial and peroxisomal dysfunction, leading to energy deficit, impaired lipid precursor supply, and ABCA1 deficiency. NMN administration counteracts this decline by rejuvenating NAD^+^‐dependent energy metabolism and promoting ABCA1‐mediated lipid efflux, thereby reinforcing a critical intestinal defense system against hepatic inflammation. It should be noted that the age‐related comparisons in this work were made between young adult (8‐week‐old) and middle‐aged (16‐month‐old) mice, which may not fully capture the more profound physiological declines in advanced age. Therefore, the observed effects and their translational potential to human aging should be interpreted within this specific developmental and aging context. Overall, these findings provide novel insights into metabolic crosstalk within the gut–liver axis and highlight the therapeutic promise of NAD^+^ precursors for mitigating age‐related hepatic pathologies.

## Author Contributions

**Yumeng Li:** wrote the manuscript and performed bioinformatics analyses. **Tongtong Bao**, **Lumin Gao**, and **Junyu Xue:** performed animal experiments. **Shujin Wang**, **Xutong Tian**, and **Caike Jin:** performed the data analysis. **Xin Wu:** conceived the work and supervised the experiments. All authors reviewed the manuscript.

## Funding

This work was supported by National Natural Science Foundation of China 32272905. Tianjin Synthetic Biotechnology Innovation Capacity Improvement Project TSBICIP‐IJCP‐004.

## Conflicts of Interest

The authors declare no conflicts of interest.

## Supporting information


**Appendix S1:** acel70445‐sup‐0001‐AppendixS1.docx.

## Data Availability

This study did not generate new unique reagents. All data reported in this paper will be shared by the lead contact upon request. This paper does not report original code. Any additional information required to reanalyze the data reported in this paper is available from the lead contact upon request.
