# Supplementary material for: Aging Triggers an Intestinal Energy Crisis and HDL3 Deficiency Disrupting Gut–Liver Axis Homeostasis
Source: Aging Cell. 2026 Mar 18;25(3):e70445. doi: 10.1111/acel.70445 (PMC13093439; doi:10.1111/acel.70445)

**Supplementary data**

Table S1: Primer sequences used in the qRT-PCR.

| genes | Primer sequence (5ʹ–3ʹ) | Accession number | Product (bp) |
| --- | --- | --- | --- |
| P53 | F: GCATGGGTCGCAGGTTCTT | NM_009877.2 | 137 |
|  | R: CATGTTCACGAAAGCCAGAGC |  |  |
| p21 | F: TCCCACTTTGCCAGCAGAATA | NM_001111099.2 | 190 |
|  | R: CAAAGTTCCACCGTTCTCGG |  |  |
| β-actin | F: CCCTCACCCAAAAG | NM_007393.5 | 165 |
|  | R: CTCAACACCTCAACCC |  |  |
| Occludin | F: CCGGCCGCCAAGGTTC | NM_008756.2 | 102 |
|  | R: CTTTCAAAAGGCCTCACGGA |  |  |
| Cludin-1 | F: TATGACCCCTTGACCCCCAT | NM_016674.4 | 132 |
|  | R: AGAGGTTGTTTTCCGGGGAC |  |  |
| ABCA1 | F: GCTCTGATGACCACCTCTGT | NM_013454.3 | 139 |
|  | R: CGCCGCTGATGAACTGAAG |  |  |
| Apoa1 | F: CGCACACACGTAGACTCTCT | NM_009692.4 | 124 |
|  | R: CCCTGGTGTGGTACTCGTTC |  |  |
| LPL | F: AAGCCCCACAAGTGTAGTCG | NM_008509.2 | 229 |
|  | R: ATAATGGGGATGCCGGTGAC |  |  |
| ANGPTL3 | F: GCTTGAGAGTCTGCTGGAAG | NM_013913.4 | 157 |
|  | R: TATGCTGTTGTCTTGCTGTTCT |  |  |

**Supplementary Figure S1.** Aging reduces peroxisomal ACOX1 expression and activity in the ileum, rescued by NMN. (a) ACOX1 protein levels in ileal tissue lysates (ELISA, normalized to total protein). (b) ACOX1 enzymatic activity measured by Amplex Red–based H₂O₂ production from C26:0-CoA. Data are presented as mean ± SEM, (n = 10). Data are express as the mean ± SEM. **p* < 0.05, ***p* < 0.01.


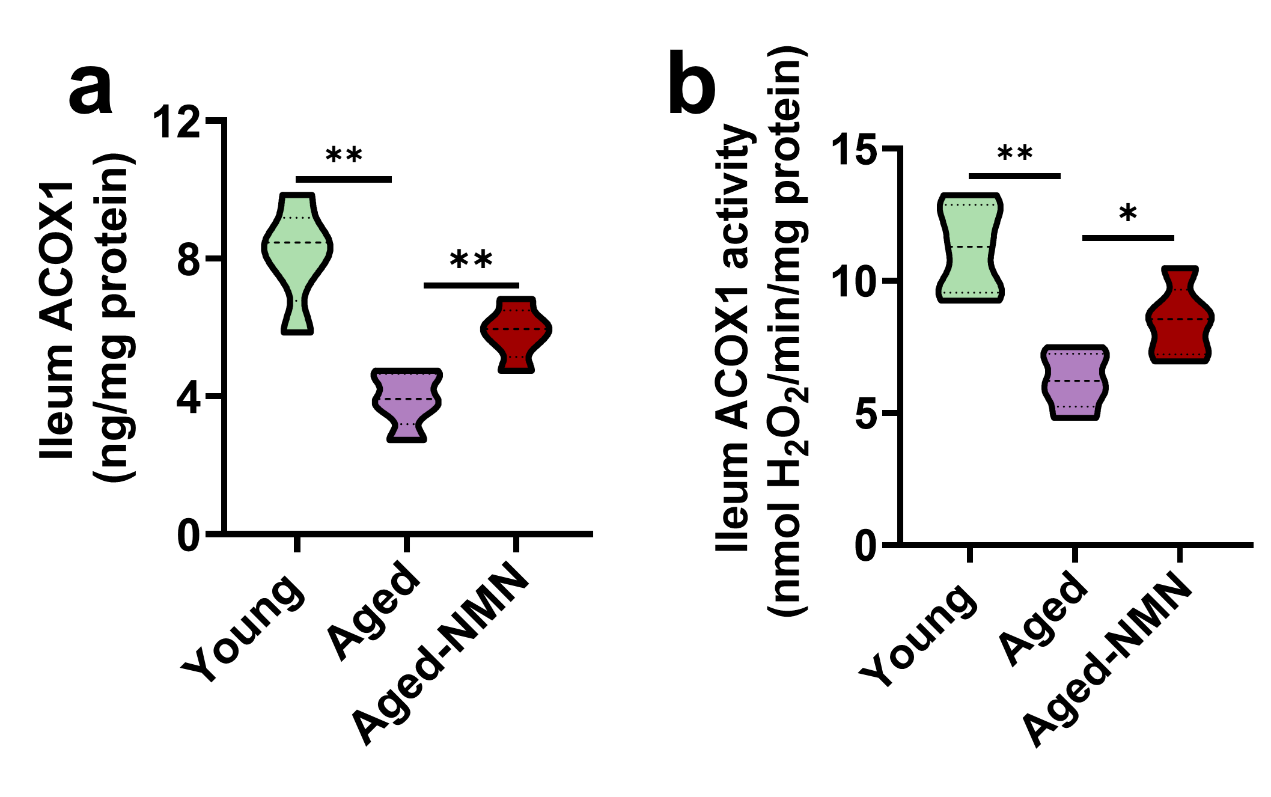

Supplement: Supplementary file 1 — Appendix S1: acel70445‐sup‐0001‐AppendixS1.docx. [file ACEL-25-e70445-s001.docx]
